# Supplementary material for: Modeling glioblastoma heterogeneity as a dynamic network of cell states
Source: Mol Syst Biol. 2021 Sep 16;17(9):e10105. doi: 10.15252/msb.202010105 (PMC8444284; doi:10.15252/msb.202010105)
Supplement: Supplementary file 6 — Source Data for Figure 5 [file MSB-17-e10105-s004.zip › Figure5A_sourcedata/GSEA_3071/hallmarks_stateVI.GseaPreranked.1623742557031/HALLMARK_APICAL_JUNCTION.html]

Details for gene set HALLMARK\_APICAL\_JUNCTION[GSEA]

|  || Dataset | state63071 |
| Phenotype | NoPhenotypeAvailable |
| Upregulated in class | na\_pos |
| GeneSet | HALLMARK\_APICAL\_JUNCTION |
| Enrichment Score (ES) | 0.29533002 |
| Normalized Enrichment Score (NES) | 1.9327452 |
| Nominal p-value | 0.007246377 |
| FDR q-value | 0.012895983 |
| FWER p-Value | 0.037 |
Table: GSEA Results Summary

  

Fig 1: Enrichment plot: HALLMARK\_APICAL\_JUNCTION      
 Profile of the Running ES Score & Positions of GeneSet Members on the Rank Ordered List

  

| PROBE | GENE SYMBOL | GENE\_TITLE | RANK IN GENE LIST | RANK METRIC SCORE | RUNNING ES | CORE ENRICHMENT || 1 | LAYN |  |  | 25 | 0.991 | 0.0258 | Yes |
| 2 | NEXN |  |  | 62 | 0.843 | 0.0436 | Yes |
| 3 | CD274 |  |  | 64 | 0.835 | 0.0709 | Yes |
| 4 | NF2 |  |  | 68 | 0.817 | 0.0970 | Yes |
| 5 | ITGA2 |  |  | 71 | 0.810 | 0.1233 | Yes |
| 6 | CALB2 |  |  | 116 | 0.711 | 0.1345 | Yes |
| 7 | FLNC |  |  | 134 | 0.685 | 0.1524 | Yes |
| 8 | ACTG2 |  |  | 142 | 0.676 | 0.1727 | Yes |
| 9 | CAP1 |  |  | 150 | 0.666 | 0.1928 | Yes |
| 10 | ITGA3 |  |  | 180 | 0.620 | 0.2051 | Yes |
| 11 | MYL9 |  |  | 195 | 0.605 | 0.2212 | Yes |
| 12 | VASP |  |  | 257 | 0.561 | 0.2227 | Yes |
| 13 | FBN1 |  |  | 311 | 0.536 | 0.2256 | Yes |
| 14 | MYH9 |  |  | 312 | 0.534 | 0.2433 | Yes |
| 15 | ZYX |  |  | 328 | 0.523 | 0.2564 | Yes |
| 16 | PFN1 |  |  | 347 | 0.513 | 0.2683 | Yes |
| 17 | CNN2 |  |  | 349 | 0.512 | 0.2849 | Yes |
| 18 | ADAMTS5 |  |  | 487 | 0.458 | 0.2618 | Yes |
| 19 | ACTG1 |  |  | 508 | 0.453 | 0.2711 | Yes |
| 20 | RRAS |  |  | 555 | 0.438 | 0.2728 | Yes |
| 21 | IRS1 |  |  | 614 | 0.421 | 0.2705 | Yes |
| 22 | NEGR1 |  |  | 639 | 0.413 | 0.2774 | Yes |
| 23 | EGFR |  |  | 669 | 0.407 | 0.2828 | Yes |
| 24 | ARPC2 |  |  | 673 | 0.406 | 0.2953 | Yes |
| 25 | ACTN1 |  |  | 845 | 0.369 | 0.2597 | No |
| 26 | SHC1 |  |  | 913 | 0.358 | 0.2528 | No |
| 27 | VCL |  |  | 935 | 0.355 | 0.2587 | No |
| 28 | CDH11 |  |  | 1024 | 0.341 | 0.2454 | No |
| 29 | TRAF1 |  |  | 1040 | 0.339 | 0.2524 | No |
| 30 | ACTB |  |  | 1243 | 0.310 | 0.2062 | No |
| 31 | TSPAN4 |  |  | 1336 | 0.300 | 0.1904 | No |
| 32 | EPB41L2 |  |  | 1343 | 0.299 | 0.1986 | No |
| 33 | BAIAP2 |  |  | 1490 | 0.281 | 0.1670 | No |
| 34 | IKBKG |  |  | 1555 | 0.274 | 0.1582 | No |
| 35 | CDK8 |  |  | 1749 | 0.255 | 0.1127 | No |
| 36 | SDC3 |  |  | 1818 | -0.252 | 0.1020 | No |
| 37 | PARD6G |  |  | 1894 | -0.263 | 0.0897 | No |
| 38 | DLG1 |  |  | 2065 | -0.290 | 0.0518 | No |
| 39 | CDH4 |  |  | 2262 | -0.326 | 0.0078 | No |
| 40 | THY1 |  |  | 2265 | -0.326 | 0.0180 | No |
| 41 | JAM3 |  |  | 2275 | -0.328 | 0.0263 | No |
| 42 | CD276 |  |  | 2308 | -0.334 | 0.0284 | No |
| 43 | PTK2 |  |  | 2347 | -0.343 | 0.0291 | No |
| 44 | KCNH2 |  |  | 2371 | -0.348 | 0.0342 | No |
| 45 | SIRPA |  |  | 2398 | -0.353 | 0.0386 | No |
| 46 | CTNNA1 |  |  | 2544 | -0.387 | 0.0108 | No |
| 47 | MMP2 |  |  | 2550 | -0.388 | 0.0222 | No |
| 48 | EXOC4 |  |  | 2669 | -0.415 | 0.0030 | No |
| 49 | TGFBI |  |  | 2679 | -0.419 | 0.0143 | No |
| 50 | ADAM23 |  |  | 2682 | -0.420 | 0.0276 | No |
| 51 | CADM3 |  |  | 2761 | -0.445 | 0.0205 | No |
| 52 | AKT3 |  |  | 2876 | -0.486 | 0.0047 | No |
| 53 | PKD1 |  |  | 2887 | -0.490 | 0.0181 | No |
| 54 | PIK3R3 |  |  | 2903 | -0.495 | 0.0303 | No |
| 55 | GAMT |  |  | 2915 | -0.500 | 0.0437 | No |
| 56 | LAMB3 |  |  | 2980 | -0.529 | 0.0433 | No |
| 57 | ARHGEF6 |  |  | 3003 | -0.544 | 0.0552 | No |
| 58 | EVL |  |  | 3032 | -0.555 | 0.0657 | No |
| 59 | GNAI2 |  |  | 3183 | -0.647 | 0.0451 | No |
| 60 | NRXN2 |  |  | 3462 | -1.034 | 0.0016 | No |
| 61 | LIMA1 |  |  | 3564 | -1.432 | 0.0207 | No |
Table: GSEA details [plain text format]

  

Fig 2: HALLMARK\_APICAL\_JUNCTION: Random ES distribution      
 Gene set null distribution of ES for **HALLMARK\_APICAL\_JUNCTION**
